# Efficient Wide-Bandgap Mixed-Cation and Mixed-Halide Perovskite Solar Cells by Vacuum Deposition

**DOI:** 10.1021/acsenergylett.0c02445

**Published:** 2021-02-03

**Authors:** Lidón Gil-Escrig, Chris Dreessen, Francisco Palazon, Zafer Hawash, Ellen Moons, Steve Albrecht, Michele Sessolo, Henk J. Bolink

**Affiliations:** † Instituto de Ciencia Molecular, Universidad de Valencia, C/Catedrático J. Beltrán 2, 46980 Paterna, Spain; ‡ Department of Physics, Karlstad University, SE-65188 Karlstad, Sweden; § Young Investigator Group for Perovskite Tandem Solar Cells, Helmholtz-Center Berlin, Kekuléstrasse 5, 12489 Berlin, Germany

## Abstract

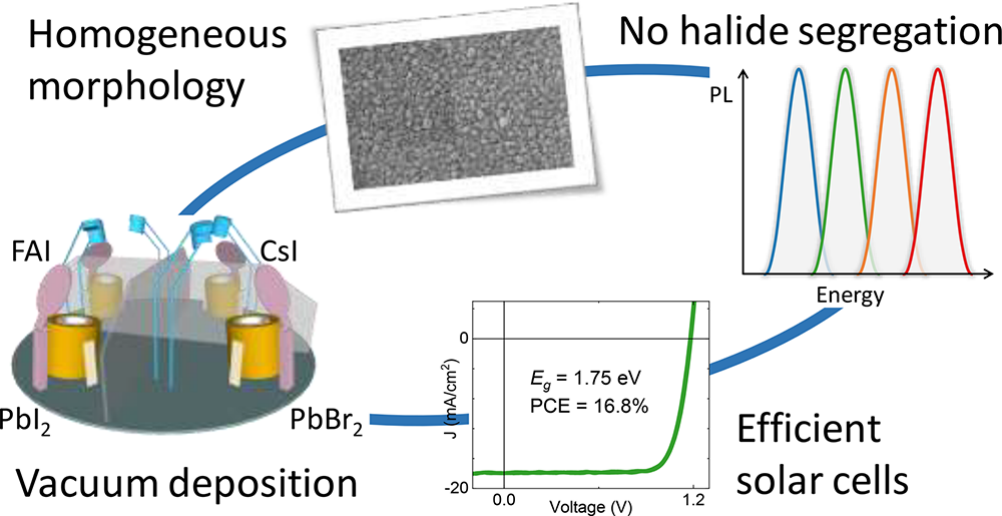

Vacuum deposition methods are increasingly applied to the preparation of perovskite films and devices, in view of the possibility to prepare multilayer structures at low temperature. Vacuum-deposited, wide-bandgap solar cells based on mixed-cation and mixed-anion perovskites have been scarcely reported, due to the challenges associated with the multiple-source processing of perovskite thin films. In this work, we describe a four-source vacuum deposition process to prepare wide-bandgap perovskites of the type FA_1–*n*_Cs_*n*_Pb(I_1–*x*_Br_*x*_)_3_ with a tunable bandgap and controlled morphology, using FAI, CsI, PbI_2_, and PbBr_2_ as the precursors. The simultaneous sublimation of PbI_2_ and PbBr_2_ allows the relative Br/Cs content to be decoupled and controlled, resulting in homogeneous perovskite films with a bandgap in the 1.7–1.8 eV range and no detectable halide segregation. Solar cells based on 1.75 eV bandgap perovskites show efficiency up to 16.8% and promising stability, maintaining 90% of the initial efficiency after 2 weeks of operation.

Among emerging photovoltaic (PV) technologies, thin-film solar cells based on organic–inorganic (hybrid) lead halide perovskites (herein called perovskites) are by far the most widely investigated. The interest toward these materials is driven by the possibility to deposit high-quality semiconducting films with simple and low-energy-demanding processes.^1−6^ This feature is a consequence of the high tolerance to defects,^7,8^ conferring perovskites with low trap density and long carrier diffusion length.^9−14^ As a result, the efficiency of single-junction solar cells has grown considerably within only a decade of development,^15^ with a record power conversion efficiency (PCE) now exceeding 25%.^16^ An important property of perovskites is the possibility to readily tune their bandgap,^17−20^ making them a suitable candidate for applications in single-junction as well as multijunction solar cells,^21−24^ e.g., in combination with narrow-bandgap absorbers such as Cu(In,Ga)Se_2_^25−28^ and silicon^29−35^ or by using complementary perovskites.^36−42^ Perovskite alloys of the type ASn_1–*x*_Pb_*x*_I_3_ (where A is an organic or inorganic cation or a mixture of them) have bandgaps in the 1.20–1.25 eV range for lead content 0.25 ≤ *x* ≤ 0.5.^43,44^ This requires perovskite compositions with wide bandgaps in the 1.75–1.85 eV range in order to aim at perovskite–perovskite tandem devices that can exceed the theoretical efficiency limit of single-junction solar cells.^21−24^ Perovskite films with wide bandgaps suitable for perovskite–perovskite tandems can be readily obtained by using mixed-iodide/bromide formulations,^17^ and mixed-A-site cations are also employed to improve the photo- and thermal stability of the compounds.^45−47^ The study of wide-bandgap perovskite materials and solar cells is a booming field of research, well summarized in recent reviews^23,48^ and in research articles containing some of the best performing devices to date.^49−51^ In comparison with narrower-bandgap materials,^52^ wide-bandgap perovskite solar cells suffer from a larger open-circuit voltage (*V*_oc_) deficit, i.e., the *V*_oc_ does not scale linearly with the bandgap as predicted by the Shockley–Queisser (SQ) limit. This deviation is due to nonradiative recombination in the perovskite bulk and at the interface with the transport layers.^51,53−55^ For this reason, a large number of studies aimed at developing bulk and surface passivation strategies as well as identifying suitable transport layers and contacts.^56−60^

The vast majority of studies on wide-bandgap perovskite solar cells relied on solution-processed perovskite thin films. Vacuum deposition is an alternative method with superior control over the film thickness and composition; it is compatible with large areas and eliminates the processing concerns related with the use of solvents.^61−63^ This is especially relevant for the fabrication of complex multilayer architectures, necessary for tandem solar cells.^37,64^ Moreover, vacuum deposition allows the deposition of pinhole-free, uniform, and smooth films.^65−68^ Early reports on vacuum-deposited wide-bandgap perovskites used the simplest formulation, methylammonium lead iodide–bromide, MAPb(I_1–*x*_Br_*x*_)_3_. We showed that this type of compound with a bandgap (*E*_g_) up to 1.7 eV (*x* ≈ 0.2) is stable even at high irradiance levels, and the corresponding perovskite solar cells exhibited a PCE up to 15.9%.^69^ When the amount of bromide is increased (*x* ≥ 0.3), the perovskite demixes into iodide- and bromide-rich phases in a process known as “halide segregation”,^45,70,71^ which can be readily monitored from the red-shifted perovskite photoluminescence (PL) spectrum.^69,72^ The iodide-rich, narrow-bandgap regions can reduce the quasi-Fermi level splitting (QFLS) and hence the maximum attainable *V*_oc_. Phase-stable hybrid perovskite films with *E*_g_ > 1.7 eV require the use of mixed-A-site cations such as cesium and formamidinium (Cs^+^, FA^+^).^45−47^ We have previously demonstrated the deposition of the wide-bandgap Cs_0.5_FA_0.4_MA_0.1_Pb(I_0.83_Br_0.17_)_3_ perovskite in a four-source cosublimation process, from PbI_2_, CsBr, formamidinium iodide (FAI), and methylammonium iodide (MAI) precursors.^73^ CsBr was used simultaneously as the source of Cs^+^ and Br^–^, a strategy later adopted by others to reduce the number of deposition sources and precursors.^74,75^ In those recent reports, FA_1–*n*_Cs_*n*_Pb(I_1–*x*_Br_*x*_)_3_ perovskites with intentionally low CsBr content (*x* ≤ 0.1) were presented, targeting perovskites with bandgaps more suitable for single-junction solar cells. In order to obtain a wide bandgap (*E*_g_ > 1.7 eV), a substantial amount of Br^–^ needs to be incorporated, hence resulting in an equally large cesium concentration. The excess of cesium was found to cause an irregular morphology and substantial bulk recombination in the perovskite, limiting the device performance.^73^

In this work, we demonstrate an alternative four-source vacuum deposition process to prepare wide-bandgap perovskites of the type FA_1–*n*_Cs_*n*_Pb(I_1–*x*_Br_*x*_)_3_ with a tunable bandgap and controlled morphology, using FAI, CsI, PbI_2_, and PbBr_2_ as the precursors. The simultaneous sublimation of the two lead halides allows the relative bromide/cesium content to be decoupled and controlled, resulting in homogeneous perovskite films with a bandgap in the 1.7–1.8 eV range and no detectable halide segregation. Solar cells based on 1.75 eV bandgap perovskites show a PCE up to 16.8% and promising stability, maintaining 90% of the initial efficiency after 2 weeks of continuous operation in inert atmosphere.

The mixed-cation lead mixed-halide perovskites were deposited by simultaneous vacuum deposition of the precursors FAI, CsI, PbI_2_, and PbBr_2_. In order to calibrate the deposition rate of each material, the specific tooling factors were determined by individually subliming them and comparing the thickness displayed from the quartz crystal microbalance (QCM) with the one measured with a mechanical profilometer. Unlike MAI, which exhibits nonstandard sublimation properties,^76^ the FAI adhesion is rather independent of the chemical composition of the surface,^77^ and hence, the FAI deposition rate can be monitored with a dedicated QCM placed nearby the corresponding thermal source. The details of the experimental conditions are provided in the Supporting Information. We prepared four perovskite compositions with increasing Br^–^/I^–^ and Cs^+^/FA^+^ ratios, with the aim to increase the bandgap while ensuring phase stability. After several variations, we found the following procedure to lead to the best performing perovskite compositions. The FAI and PbI_2_ deposition rates were kept constant at 0.8 and 1 Å/s, respectively. The PbBr_2_ deposition rate was varied from 0.07 to 0.22 Å/s, while the CsI rate was increased from 0.25 to 0.45 Å/s, to prevent halide segregation in the bromide-rich formulations. The substrates were kept at room temperature (RT) during deposition, and the films were not annealed and used as-deposited.

The absorbance spectra of a series of 500 nm thick wide-bandgap FA_1–*n*_Cs_*n*_Pb(I_1–*x*_Br_*x*_)_3_ perovskite films on glass substrates are reported in Figure 1a. All films show the expected perovskite absorption profile, with absorbance >1 for wavelengths below approximately 550 nm. With increasing PbBr_2_ content (deposition rate), we observed the expected blue-shift of the absorption cutoff from approximately 740 to 690 nm, indicating that indeed bromide is incorporated into the perovskite structure. The corresponding Tauc plots (Figure 1b) allow the bandgap energy of the four perovskite compositions to be estimated, with *E*_g_ increasing steadily from 1.70 to 1.77 eV. The bulk Cs^+^ (*n*) and Br^–^ (*x*) concentrations in the different FA_1–*n*_Cs_*n*_Pb(I_1–*x*_Br_*x*_)_3_ samples were estimated by energy dispersive X-ray spectroscopy (EDS). As highlighted in Figure 1c, bromide and cesium contents were found to be in the 0.14 ≤ *x* ≤ 0.30 and 0.26 ≤ *n* ≤ 0.39 ranges, respectively. The Cs^+^ concentration was adjusted in order to stabilize the perovskite formulations against halide segregation, in particular when the bromide content is increased to obtain the wider-bandgap materials. In Figure S1, the photoluminescence (PL) spectra of perovskite films with a 1.75 eV bandgap (*x* = 0.27) and varying amounts of Cs+ (*n*) are presented, showing that the perovskite is photostable for CsI deposition rates >0.3 Å/s (*n* > 0.3, see discussion in the Supporting Information and Figure S2). For this particular perovskite (*E*_g_ = 1.75 eV), we have performed further compositional analysis by using high-resolution X-ray photoemission spectroscopy (HR-XPS) to measure the core levels of the perovskite elements. The collected spectra were consequently normalized, using the corresponding atomic sensitivity factors, to find the surface atomic composition.^78^ The surface contents of bromide and cesium using HR-XPS were found to be *x* = 0.24 and *n* = 0.40, respectively (Figure S3), only slightly different from the values obtained by EDS. Taking the difference in information depth into consideration for XPS^79^ (only a few nanometers) and EDS^80^ (several hundreds of nanometers), the results imply that there is no substantial compositional difference between the bulk and the surface of the perovskite films. In Figure 1d, the PL spectra of the entire series of stabilized perovskite films is reported. Spectra are collected over time under continuous wave laser illumination (515 nm), at an irradiance of approximately 300 mW/cm^2^, corresponding to a 5–6 sun of equivalent intensity. Note that after an initial drop of the PL intensity in the first 15–20 min, we found it to be stable for up to 1 h of continuous measurement (Figure S4). Even with these harsh conditions, we did not observe any low-energy PL components, which would indicate halide segregation into iodide-rich regions. Note that a semilogarithmic scale is used for all spectra in Figure 1d to highlight the persistence of a single PL component. The differences in line width are due to varying signal-to-noise ratios among the different samples, hence the full width at half-maximum (fwhm) measured by a Gaussian fit is reported for reference. We observed photoinduced halide segregation only when the bromide content *x* was increased to 0.4 (Figure S5), indicating that the perovskite formulation should be substantially modified in order to obtain phase-stable materials with higher bromide content. We have also measured the PL spectra for a perovskite film on glass, exciting the sample both from the glass and from the perovskite side. As shown in Figure S6, the spectral shape and position is unaltered, suggesting that there are no obvious compositional changes through the cross section of the film. The lower PL intensity observed when shining the laser directly on the perovskite film indicates a larger degree of nonradiative recombination at the perovskite surface. In order to estimate the reproducibility of the deposition process, we compared the PL spectra for films obtained from seven consecutive deposition runs. As depicted in Figure S7, the bandgap variation for seven different batches of the perovskite with a bandgap of 1.75 eV is only 17 meV, demonstrating the good reproducibility of the vacuum deposition process.

**Figure 1:**
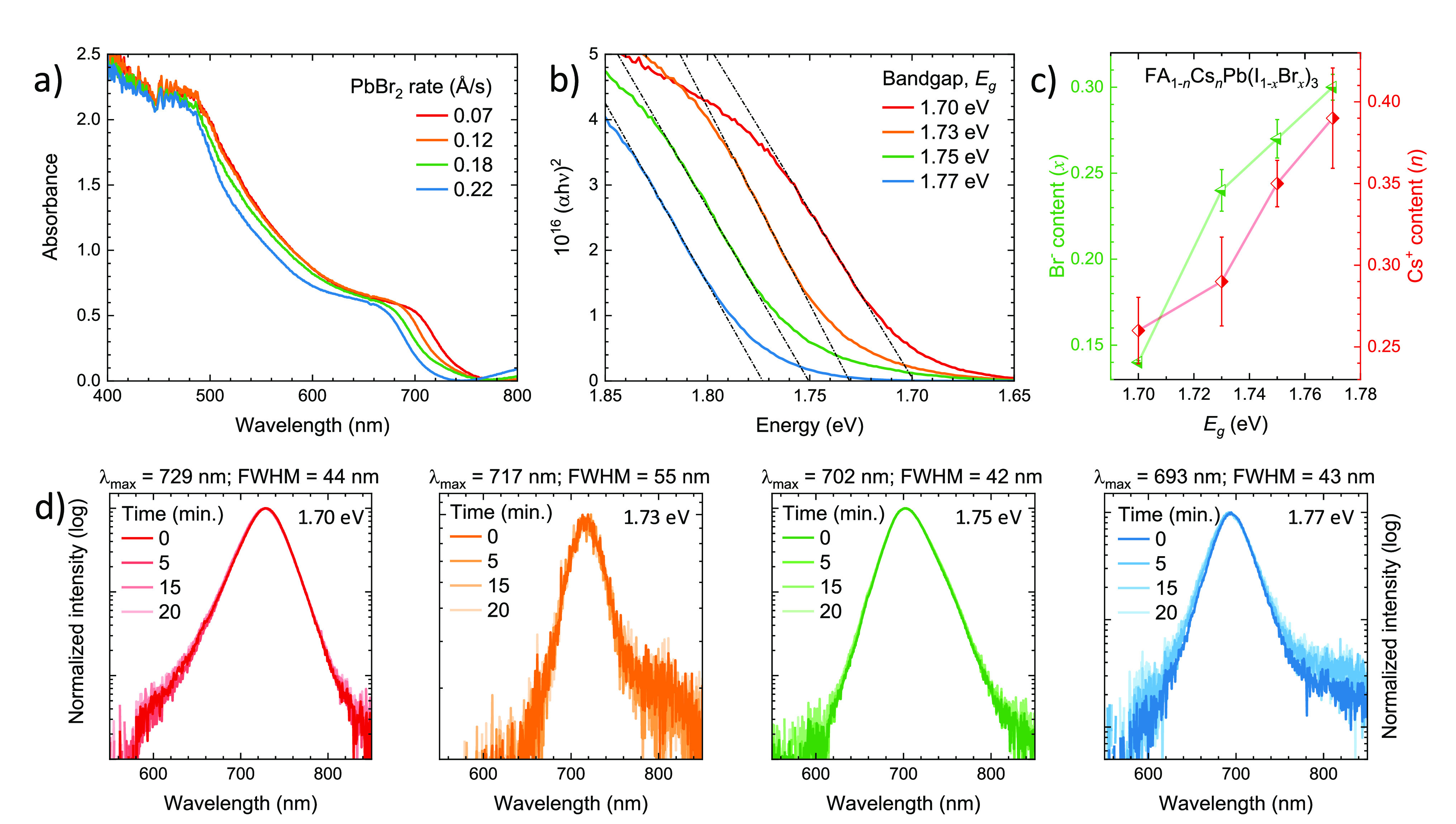
(a) Absorbance spectra of a series of FA_1–*n*_Cs_*n*_Pb(I_1–*x*_Br_*x*_)_3_ perovskite films obtained with increasing PbBr_2_ deposition rate and (b) corresponding Tauc plot and estimated bandgap energies (*E*_g_). The film thickness is 500 nm for all samples. (c) Bulk bromide (left, green) and cesium (right, red) content in the perovskite films estimated by energy dispersive X-ray spectroscopy (EDS). Error bars are the standard deviation of measurements obtained from films of different deposition runs. (d) Normalized photoluminescence (PL) spectra of the same samples recorded over time (up to 20 min) under continuous illumination. The excitation source is a green laser (515 nm) with an irradiance of approximately 300 mW/cm^2^.

The perovskite films were further analyzed by X-ray diffraction (XRD, Figure 2a). The XRD data can be fitted considering a single distorted perovskite phase in combination with a marginal contribution from PbI_2_, which is mostly visible by its main peak around 2θ = 12.8°. The perovskite phase considered here corresponds to the space group *Pnma* (orthorhombic system), which is the reported stable phase of CsPbBr_3_ at room temperature as well as of MAPbBr_3_ at low temperature (see Inorganic Crystal Structure Database, ICSD references #243735 and #158306). This can be considered a lower-symmetry derivative (hettotype) of the highest-symmetry cubic perovskite (aristotype; space group *Pm*-3m), where PbX_6_ octahedra are slightly tilted (see scheme in Figure S8).^81^ All samples show a clear preferential orientation along the *b*-axis (perpendicular to the substrate), as evidenced by the two main reflections at 2θ = 14.2° and 2θ = 28.7°, which are ascribed to the (020) and (040) planes. The unit cell volumes derived from the whole-pattern Le Bail fits presented in Figure 2a are plotted in Figure 2b, showing a clear shrinkage of the unit cell from 831.5 to 821 Å^3^ as more iodide anions are replaced by bromide anions, which have a smaller ionic radius (as well as FA^+^ being replaced by Cs^+^, though this replacement typically has a smaller effect in the lattice expansion or shrinkage than the anion exchange). The different values obtained by the whole-pattern fits are the result of small shifts of the XRD peaks, which can be better visualized in Figure S9.

**Figure 2:**
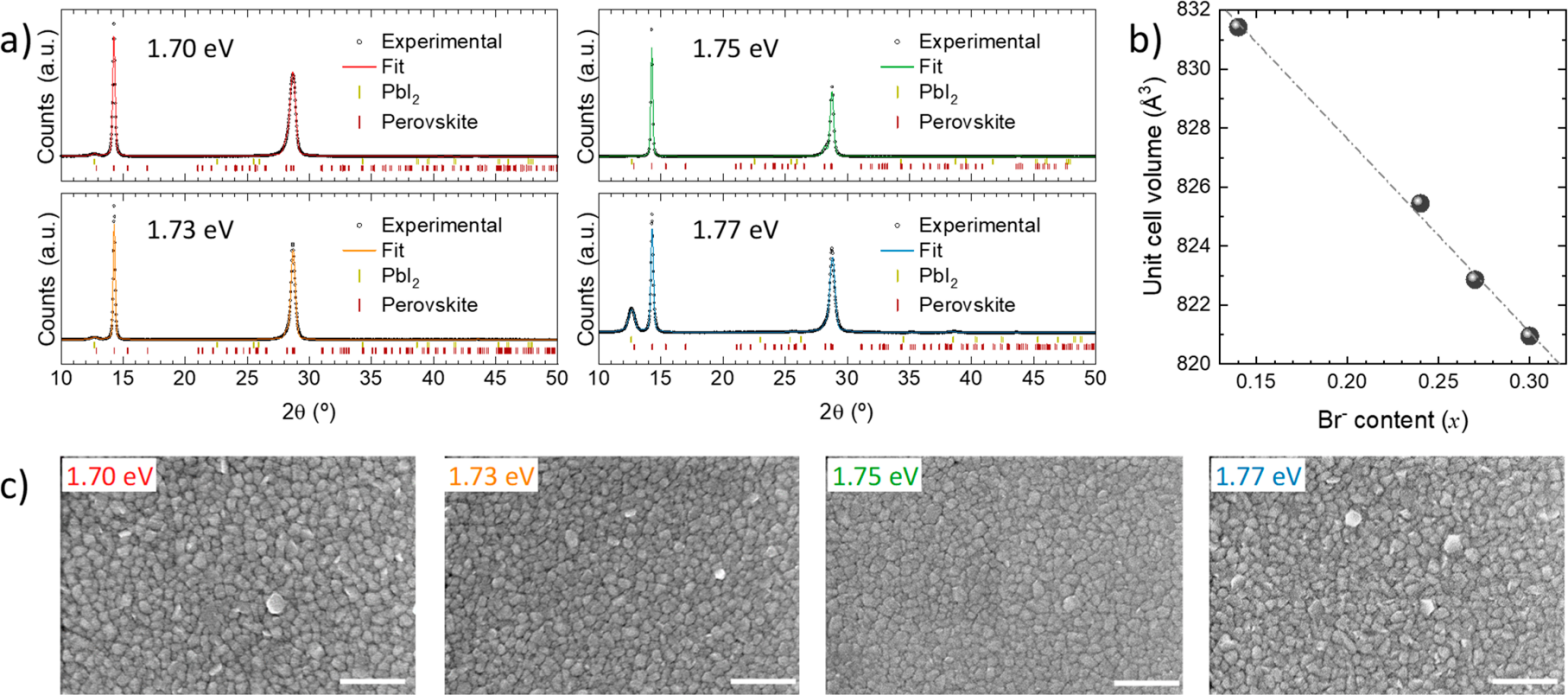
(a) Whole-pattern Le Bail fit (colored lines) of XRD patterns (open black circles). Vertical markers correspond to calculated Bragg’s reflections for a distorted perovskite phase (dark red) and PbI_2_ (yellow). (b) Calculated unit cell volumes as obtained from fit as a function of the bromide content estimated by EDS. (c) Surface morphology of the whole sample series as observed by SEM; scale bar corresponds to 500 nm.

We also studied the morphology of the FA_1–*n*_Cs_*n*_Pb(I_1–*x*_Br_*x*_)_3_ thin films by SEM top view images (Figure 2c). All films exhibit a similar surface morphology, composed of small grains (typical size in the 50–100 nm range) arranged in a compact and homogeneous manner. Such small grains are a common feature of vacuum-deposited perovskite films, as highlighted in several previous reports.^82−84^ The morphology of these materials is in stark contrast with what we have observed before for vacuum-deposited Cs_0.5_FA_0.4_MA_0.1_Pb(I_0.83_Br_0.17_)_3_ films, obtained using CsBr as a simultaneous precursor for Cs^+^ and Br^–^.^73^ As discussed in the introduction, that process does not allow to the contents of the two ions (Cs^+^ and Br^–^) to be separately fine-tuned, leading to irregular morphology with randomly oriented grains growing on the perovskite surface.

To shortly summarize, we demonstrated the successful room temperature deposition of highly oriented FA_1–*n*_Cs_*n*_Pb(I_1–*x*_Br_*x*_)_3_ films with homogeneous morphology and controlled and tunable bandgap from 1.70 to 1.77 eV. This is achieved with a four-source vacuum deposition process, using FAI, CsI, PbI_2_, and PbBr_2_ as the precursors. The use of PbBr_2_ and CsI is important to control the relative bromide/cesium content in each sample, which is found to be stable against photoinduced halide segregation. In view of the favorable properties of the perovskite films presented above, we used them as the light-absorbing layer in fully vacuum-deposited perovskite solar cells. We fabricated p–i–n solar cells with the structure reported in Figure S10. Briefly, patterned indium tin oxide (ITO) transparent electrodes were coated with MoO_3_ (5 nm) to enhance hole transfer between ITO and the hole transport layer (HTL), a 10 nm thick film of *N*4,*N*4,*N*4″,*N*4″-tetra([1,1′-biphenyl]-4-yl)-[1,1′:4′,1′′-terphenyl]-4,4′′-diamine (TaTm, 10 nm). Afterward, a 500 nm thick perovskite film was deposited on top and capped with an electron transport layer (ETL, C_60_, 25 nm). A thin (8 nm) film of bathocuproine (BCP) was used to ensure ohmic contact in between the ETL and a silver electrode (100 nm thick). Further details of the solar cell fabrication are reported in the Supporting Information.

The external quantum efficiency (EQE, Figure 3a) spectra were found to be similarly high (in the 0.8–0.9 range) for the four materials through the whole visible spectrum. The onset of the spectral response in the low-energy regime follows the trend expected from the perovskites’ optical absorption and bandgap (Figure 1), i.e., the EQE onset shifts to lower wavelengths when the content of bromide is increased. The corresponding short-circuit currents (*J*_sc_), calculated by integration of the EQE over the global AM1.5G solar spectra, decrease from 18.3 to 16.5 mA cm^–2^ when widening the bandgap from 1.70 to 1.77 eV. These values agree with those extracted from current-density vs voltage (*J*–*V*) curves under simulated solar illumination, depicted in Figure 3b. The characteristic PV parameters are reported in Table 1. All solar cells showed a high fill factor (FF, between 76 and 80% on average), indicating an efficient charge extraction of the photogenerated charge carriers. We also observed negligible hysteresis in between the forward and reverse scans, which suggests that either ion migration or interface recombination (or both) are suppressed in these perovskite solar cells.^85,86^ More interesting is the trend of the measured *V*_oc_, which scales with the perovskite bandgap (Figure 3c), going from 1.14 V for the 1.70 eV absorber to 1.21 V for the largest 1.77 eV bandgap, on average. The corresponding *V*_oc_ deficit, defined as (*E*_g_/*q* – *V*_oc_), was found to be rather large (0.56–0.58 V) and constant through the series of devices, indicating a common origin of the nonradiative recombination channels. Although far from the radiative limit for these semiconductors (see discussion below), these values are the highest voltages obtained for wide-bandgap mixed-cation/halide perovskite solar cells deposited by vacuum deposition.

**Figure 3:**
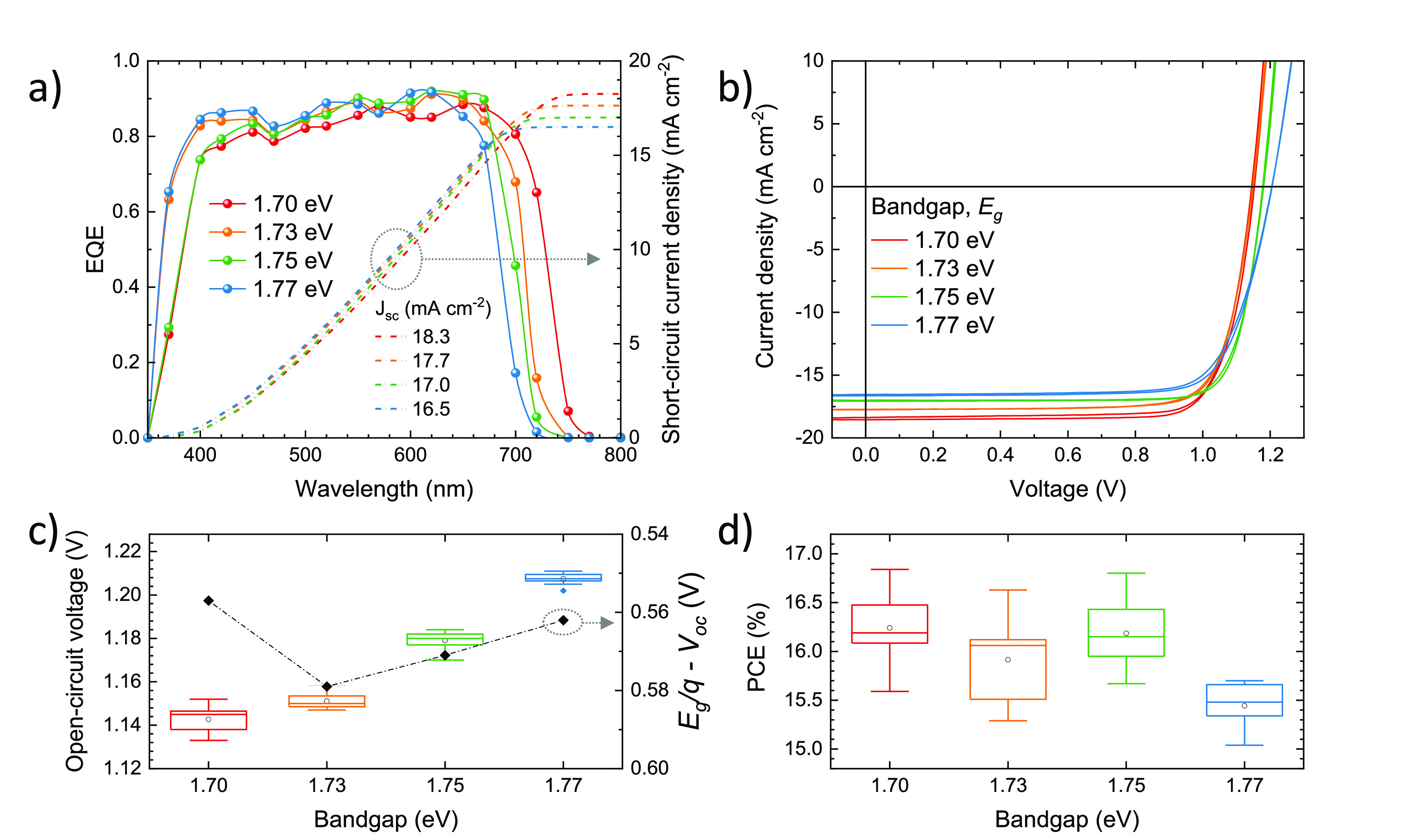
Characterization of wide-bandgap perovskite solar cells with FA_1–*n*_Cs_*n*_Pb(I_1–*x*_Br_*x*_)_3_ absorbers in a p–i–n configuration. (a) External quantum efficiency (EQE) spectra (line and symbols) and corresponding short-circuit current-density (*J*_sc_, dotted lines) calculated by integration of each EQE spectrum with the global AM1.5G solar spectrum. (b) *J*–*V* curves under simulated solar illumination recorded in forward (from short to open circuit) and reverse (from open to short circuit) bias for representative pixels. Summary of the (c) open-circuit voltage (*V*_oc_) and (d) power conversion efficiency (PCE) measured for perovskite solar cells as a function of the bandgap determined from Tauc analysis. In the right axis in (c), the open-circuit voltage deficit (*E*_g_/*q* – *V*_oc_) is also reported (lines are guides to the eye).

**Table 1:** Average Photovoltaic Parameters with Standard Deviation Extracted from *J*–*V* Curves under Simulated Solar Illumination from Wide-Bandgap Perovskite Solar Cells with FA_1–*n*_Cs_*n*_Pb(I_1–*x*_Br_*x*_)_3_ in p–i–n Configuration^a^

composition	*E*_g_ (eV)	*J*_sc_ (mA cm^–2^)	FF (%)	*V*_oc_ (mV)	PCE (%)
FA_0.74_Cs_0.26_Pb(I_0.86_Br_0.14_)_3_	1.70	18.3 ± 0.2	77.5 ± 0.9	1142 ± 6	16.2 ± 0.3
FA_0.71_Cs_0.29_Pb(I_0.76_Br_0.24_)_3_	1.73	17.7 ± 0.1	77.5 ± 1.2	1151 ± 3	15.9 ± 0.4
FA_0.65_Cs_0.35_Pb(I_0.73_Br_0.27_)_3_	1.75	17.0 ± 0.1	80.3 ± 0.8	1179 ± 4	16.2 ± 0.3
FA_0.61_Cs_0.39_Pb(I_0.70_Br_0.30_)_3_	1.77	16.6 ± 0.1	76.1 ± 0.8	1208 ± 2	15.5 ± 0.2

a At least 12 cells for each bandgap have been tested.

Overall, our wide-bandgap vacuum-deposited perovskite solar cells show PCEs of about 16% for bandgaps in the 1.70–1.75 eV range (Figure 3d). The best pixels were obtained for the wide-bandgap perovskite with *E*_g_ = 1.75 eV, with a PCE up to 16.8% (Figure S11). The reduction in efficiency observed for the solar cells with the highest bromide content are partially expected due to the increased bandgap (1.77 eV), although a small decrease in FF also contributes to the efficiency reduction.

In order to further assess the quality of the wide-bandgap FA_1–*n*_Cs_*n*_Pb(I_1–*x*_Br_*x*_)_3_ perovskites and the corresponding solar cells, we investigated their EQE response in the bandgap region. From the semilogarithmic plot in Figure 4a, one can see for all devices a steep drop of the EQE around the perovskite’s bandgap.

**Figure 4:**
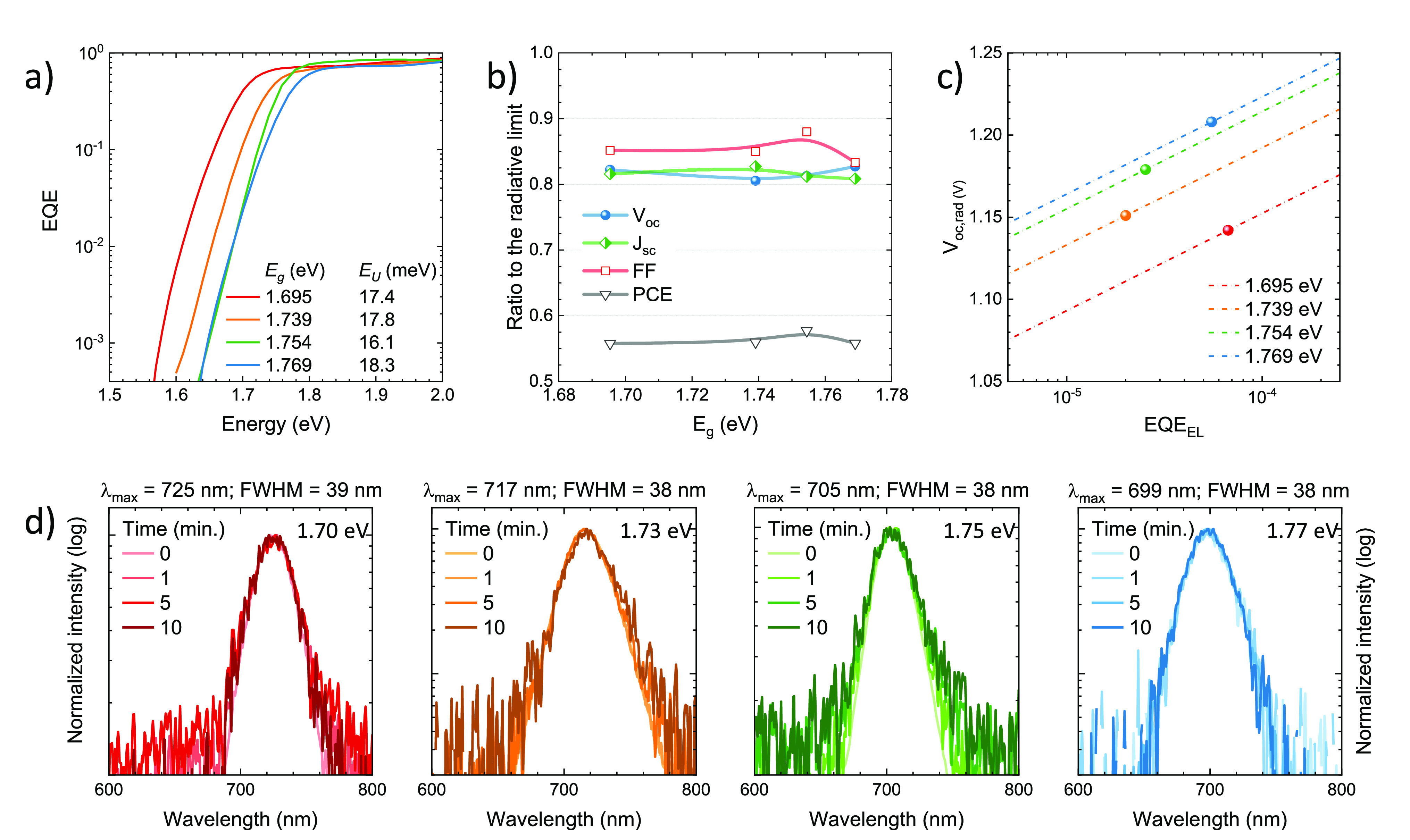
Optoelectronic analysis of FA_1–*n*_Cs_*n*_Pb(I_1–*x*_Br_*x*_)_3_ solar cells with different bandgaps. (a) Sensitive EQE spectra in the bandgap region and calculated bandgap and Urbach energies. (b) Ratio of measured *V*_oc_, *J*_sc_, and FF to their maximum theoretical (radiative) limit (lines are guides to the eye). (c) Relation between the open-circuit voltage in the radiative limit with the electroluminescence quantum efficieny, EQE_EL_.of a solar cell (lines) for the four bandgaps studied here. Symbols shows the measured *V*_oc_, allowing the corresponding EQE_EL_ to be estimated. (d) Electroluminescence (EL) spectra of the same samples recorded over time (up to 10 min) under continuous forward bias. Each cell was driven with a constant current-density equal to the *J*_sc_ obtained under simulated solar illumination.

From the slope, we extracted the Urbach energies (*E*_U_, see Supporting Information for details), which are in the range of 16–18 meV, indicating a low electronic disorder that is essential to obtain high *V*_oc_.^87^ The bandgaps obtained from the derivative of the sensitive EQE measurements (Figure 4a) agree well with the values estimated from the Tauc plots in Figure 1b.^88^ To be able to compare the limitations in the performance of absorbers with different bandgaps with each other, we divided the measured key performance indicators as obtained from the *J–V* curves by their maximal obtainable values in Figure 4b. The radiative limit of the *V*_oc_ (V_oc,rad_) was calculated via the EQE response,^89^ while the FF and *J*_sc_ were obtained directly from detailed balance calculations given the specific bandgap of each material (SQ limit).^90,91^ In general, all device parameters were found to be approximately at 80–90% of the theoretical maxima, highlighting the high quality of the perovskite films and devices reported here. The FF shows the highest ratio of the three parameters, indicating good rectification and low series resistance. We noted that the solar cells prepared with the 1.75 eV perovskite showed consistently higher FF, which might originate from a larger charge carrier mobility for this particular composition. The *J*_sc_ and *V*_oc_ more severely limit the overall performance, with ratios only slightly above 80%. Interestingly, the *V*_oc_/V_oc,rad_ ratio rises marginally with the bandgap, in contrast to the commonly observed behavior. This behavior might be related to the increased amount of PbI_2_ in the wider-bandgap material as seen from XRD, which has been reported to passivate trap states in perovskite films.^92,93^ However, the EQE of the electroluminescence (EQE_EL_), estimated from the obtained photovoltage (Figure 4c, EQE_EL_ = exp(*V*_oc_ – *V*_oc,rad_)/*kT*)), is in the 10^–5^ to 10^–4^ range for all devices, indicating the presence of nonradiative recombination either in the perovskite bulk or at the interface with the transport layers.^53−55^ In order to shed light on the more relevant type of recombination, we evaluated the PL intensity of a perovskite film with and without the charge transport layers. As shown in Figure S13, the transport layers do quench the perovskite luminescence, indicating the presence of interface recombination. However, if we consider the relative PLQY obtained by integrating the PL spectra and normalizing it to the PL of the bare perovskite, we can estimate the QFLS difference in the presence of the transport materials. The difference in QFLS between the full stack (TaTm/perovskite/C_60_) and a bare perovskite film is only about 50 mV. From this observation and taking into account that the *V*_oc_ of a device is about 300 mV lower compared to its radiative limit, we can conclude that the *V*_oc_ is mainly limited by nonradiative recombination in the bulk of the perovskite layer. Hence, future efforts should be directed toward passivation of bulk defects, through the use of additives or by modulating the deposition process. A possible loss pathway is the formation of iodide-rich domains driven by currents and electric fields, which would reduce the QFLS and hence the maximum attainable *V*_oc_. It has been reported that even perovskite compositions that are stable under illumination can show halide segregation under current injection, which is evidenced by the EL spectrum of the diodes.^94^ Hence, we tested our series of perovskite solar cells in forward bias, applying a current-density equivalent to their *J*_sc_, and recorded the EL spectra as a function of time. Measurements are taken for up to 10 min as the EL intensity decreases over time (Figure S14) so that after 10–15 min, the spectrometer cannot resolve the spectra anymore due to the low signal-to-noise ratio. As highlighted in Figure 4d, the wide-bandgap solar cells were found to be very stable also under current injection, with the EL spectra showing a single component and no spectral changes over time. The EL spectral positions are also in agreement with the PL signals depicted in Figure 1d. Therefore, we exclude halide segregation as a main loss factor.

We finally evaluated the stability of the most efficient solar cells based on FA_1–*n*_Cs_*n*_Pb(I_1–*x*_Br_*x*_)_3_ perovskites with *E*_g_ = 1.75 eV. Both the shelf life (in the dark) and the operational stability under illumination were evaluated. The devices were encapsulated with a UV-curable resin and a glass slide, and the stability was evaluated in a nitrogen atmosphere to minimize the effect of environmental factors on the degradation (note that no differences in performance were observed after encapsulation, see Figure S15). For the shelf life stability, the *J–V* characteristics under 1 sun illumination were recorded periodically at room temperature (Figure 5a).

**Figure 5:**
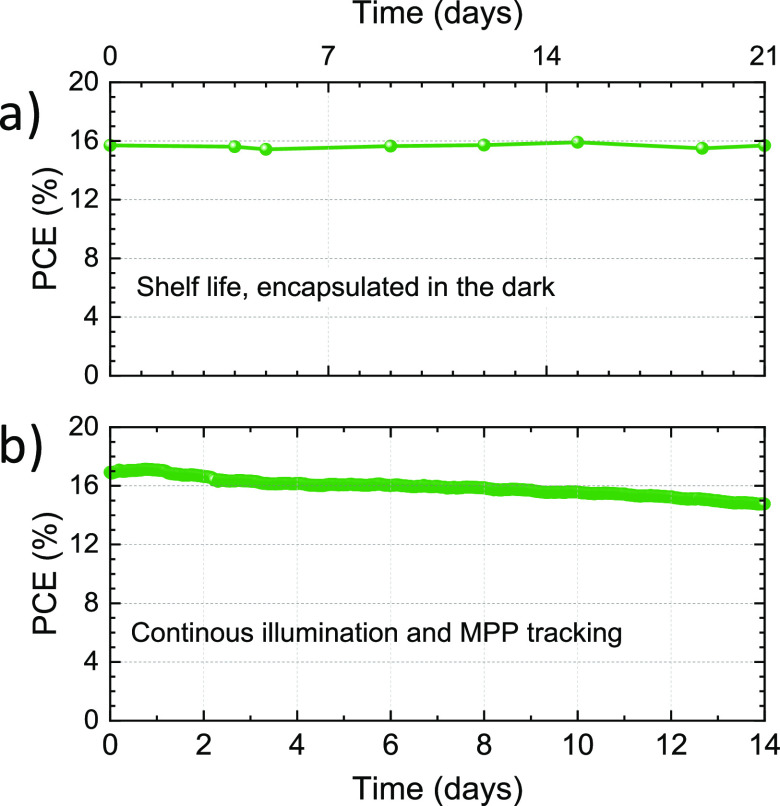
Stability assessment of wide-bandgap FA_0.65_Cs_0.35_Pb(I_0.73_Br_0.27_)_3_ perovskite solar cells with *E*_g_ = 1.75 eV, performed on encapsulated devices in a nitrogen atmosphere. (a) Shelf life measurements for devices kept in the dark. (b) Constant maximum power point tracking under continuous illumination.

After 500 h of storage (3 weeks), the PCE was found unvaried from the initial value, indicating an overall good stability of the perovskite film within the device structure used here. To evaluate the operational stability, the devices were maintained at their maximum power point (MPP) under a simulated 1 sun illumination with white LEDs at RT (25 °C) in nitrogen (Figure 5b). Under these operational conditions, the solar cell exhibited a remarkable stability, maintaining 90% of the initial PCE after 340 h (more than 2 weeks) of continuous operation.

In summary, we showed the room temperature preparation of wide-bandgap perovskite films of the type FA_1–*n*_Cs_*n*_Pb(I_1–*x*_Br_*x*_)_3_ by thermal vacuum deposition. The simultaneous sublimation of four precursors and in particular the use of PbBr_2_ and CsI to individually control the bromide and cesium content allows the deposition of wide-bandgap perovskites with bandgaps between 1.7 and 1.8 eV. In this way, no signatures of halide segregation and an overall homogeneous morphology can be attained. These film properties translate into efficient p–i–n solar cells, with photovoltaic parameters at 80% of their maximum theoretical (radiative) limits, highlighting the high quality of the as-deposited perovskite semiconductors. We obtained solar cells with a bandgap of 1.75 eV and power conversion efficiency up to 16.8%. These devices retain 90% of their initial efficiency after more than 2 weeks of continuous operation. This work opens up the way toward the vacuum processing of photostable wide-bandgap perovskite solar cells for integration in tandem devices.

## Supporting Information Available

Experimental methods, photoluminescence spectra analysis, XPS analysis, details of structural XRD analysis, device layout, details of calculation of bandgap, Urbach energy and radiative limit of the *V*_oc_ from EQE measurements, time-dependent electroluminescence intensity (PDF)
