# Supplementary material for: Efficient Wide-Bandgap Mixed-Cation and Mixed-Halide Perovskite Solar Cells by Vacuum Deposition
Source: ACS Energy Lett. 2021 Feb 3;6(2):827–36. doi: 10.1021/acsenergylett.0c02445 (PMC8461651; doi:10.1021/acsenergylett.0c02445)
Supplement: Supplementary file 1 — nz0c02445_si_001.pdf [file nz0c02445_si_001.pdf]

SUPPORTING INFORMATION

Efficient wide-bandgap mixed-cation and mixed-halide perovskite solar cells by vacuum deposition

Lidón Gil-Escrig,[a] Chris Dreessen,[a] Francisco Palazon,[a] Zafer Hawash,[b] Ellen Moons,[b] Steve Albrecht,[c] Michele Sessolo [a,]* and Henk J. Bolink[a]

[a]*Instituto de Ciencia Molecular, Universidad de Valencia, C/ Catedrático J. Beltrán 2, 46980, Paterna, Spain. E-mail:* michele.sessolo@uv.es

[b]*Department of Physics, Karlstad University, SE-65188 Karlstad, Sweden*

[c]*Young Investigator Group for Perovskite Tandem Solar Cells, Helmholtz-Center Berlin, Kekuléstrasse 5, 12489 Berlin, Germany*

Materials. *N4,N4,N4',N4'*-tetra([1,1'-biphenyl]-4-yl)-[1,1':4',1'-terphenyl]-4,4'-diamine (TaTm) was provided by Novaled GmbH and fullerene (C_{60}) was purchased from Merck KGaA. PbI_2, CH_3NH_3I (MAI), MoO_3, and bathocuproine (BCP) were purchased from Luminescence Technology Corp. $CHNH_2NH_2I$ (FAI) was purchased from Greatcell Solar. $PbBr_2$ was obtained from Tokyo Chemical Industry. All materials were used as received.

Device preparation. ITO-coated glass substrates were subsequently cleaned with soap, water and isopropanol in an ultrasonic bath, followed by 20 min UV-ozone treatment. The substrates were transferred to a vacuum chamber integrated in a nitrogen-filled glovebox and evacuated to a pressure of 10^{-6} mbar for the charge extraction layers' deposition. In general, the deposition rate for the TaTm and C_{60} was 0.5 Å/s while the thinner BCP layer was sublimed at 0.2 Å/s. MoO_3 and Ag were deposited in a second vacuum chamber using aluminum boats as sources, by applying currents ranging from 2.0 to 4.5 A. The perovskite was evaporated in another vacuum chamber, equipped with four evaporation sources (Creaphys) and with independent temperature controllers and shutters. All sources have a dedicated QCM sensor above, and an additional one is installed close to the substrates for the overall deposition rate measurement. All sources were individually calibrated for their respective materials and no cross-reading between different materials is ensured by the position of the sources, shutters, sensors. During the $FA_{1-n}Cs_nPb(I_{1-x}Br_x)_3$ perovskite deposition, the FAI and PbI_2 deposition rates were kept constant at 0.8 Å/s and 1 Å/s, respectively. In order to tune the bandgap while ensuring phase stability, $PbBr_2$ and CsI

were sublimed at varying deposition rates, in the range of 0.25-0.45 Å/s and 0.07-0.22 Å/s, respectively. During the perovskite deposition, the pressure of the chamber was mantained at $8 \cdot 10^{-6}$ mbar and the substrates were kept at room temperature. Typical sublimation temperatures for the precursors were 150 ºC for FAI, 310 ºC for PbI_2, 280-300 ºC for $PbBr_2$ and 490 - 520ºC for CsI.

Characterization. Absorption spectra were collected using fiber optics based Avantes Avaspec2048 Spectrometer. The photoluminescence spectra were measured with an Avantes Avaspec2048 spectrometer and films were illuminated with a diode laser of integrated optics, emitting at 522 nm. All the spectra were collected with an integration time of 1 s. The crystalline structure of the powder and film samples was studied by X-ray diffraction (XRD). The patterns were collected in Bragg-Brentano geometry on an Empyrean PANalytical powder diffractometer with a copper anode operated at 45 kV and 40 mA. Further analysis including Le Bail fits were performed with Fullprof software. Scanning Electron Microscopy (SEM) images were performed on a Hitachi S-4800 microscope operating at an accelerating voltage of 2 kV over platinum-metallized samples.

High resolution x-ray photoemission spectroscopy (XPS, Sceinta Omicron SES-100, non-monochromatic Al−Kα = 1486.6 eV) measurements were performed to quantify the chemical composition of the top surface of the perovskite film. The peak fitting and atomic percentage calculations were performed using CasaXPS 2.3.16 software. Shirley background lines and gaussian-lorentzian shape lines were used for fitting the components. The binding energy (BE) for XPS was calibrated by measuring Fermi edge (E_F = 0 eV) and Au-4$f_{7/2}$ (84.0 eV) on a clean Au surface. The x-ray gun was operated at 250W. The estimated energy resolution of XPS is 0.7 eV. XPS measurements were performed in an ultra-high vacuum (UHV) chamber with pressure of about 1×10^{-10} mbar. The acquisition was performed with pass energy of 20 eV and dwell time of 0.1 sec. X-ray-induced sample damage was monitored by taking several consecutive spectra and comparing those spectra. In case of no damage observed, all the collected spectra for each core level were averaged into the final high resolution spectrum. Time acquisition for each scan varied from 40 to 70 sec depending on the core level region. To minimize any possible unnecessary x-ray exposure time, special care was taken while optimizing and acquiring XPS measurements. No X-ray-induced damage was observed on the films. All the calculated atomic percentages are normalised according to Hartree-Slater Subshell Photoionization Cross-Sections at 1486.6 eV.[1]

The J-V curves for the solar cells were recorded using a Keithley 2612A SourceMeter in a -0.2 and 1.3 V voltage range, with 0.01 V steps and integrating the signal for 20 ms after a 10 ms delay, corresponding to a scan speed of about 0.3 Vs^{-1}. The devices were illuminated under a

Wavelabs Sinus 70 LED solar simulator. The light intensity was calibrated before every measurement using a calibrated Si reference diode. The external quantum efficiency (EQE) was estimated using the cell response at different wavelength (measured with a white light halogen lamp in combination with band-pass filters), where the solar spectrum mismatch was corrected with a calibrated Silicon reference cell (MiniSun simulator by ECN, from Netherlands).

For the sensitive EQE measurements, the cell was illuminated by a Quartz-Tungsten-Halogen lamp (Newport Apex 2-QTH) through a monochromator (Newport CS130-USB-3-MC), a chopper at 279 Hz and a focusing lens. The device current was measured as a function of energy from 2.1 eV to 1.2 eV in 0.02 eV steps using a lock-in amplifier (Stanford Research Systems SR830). The system was calibrated and the solar spectrum mismatch was corrected using a calibrated Silicon reference cell.

Solar cell stability measurements were recorded using a maximum power point tracker (mppt) system, with a white LED light source under 1 sun equivalent, developed by Candlelight. During the mppt measurements, a flow of N_2 gas was used and temperature was kept at 300 K using a water-circulating cooling system.

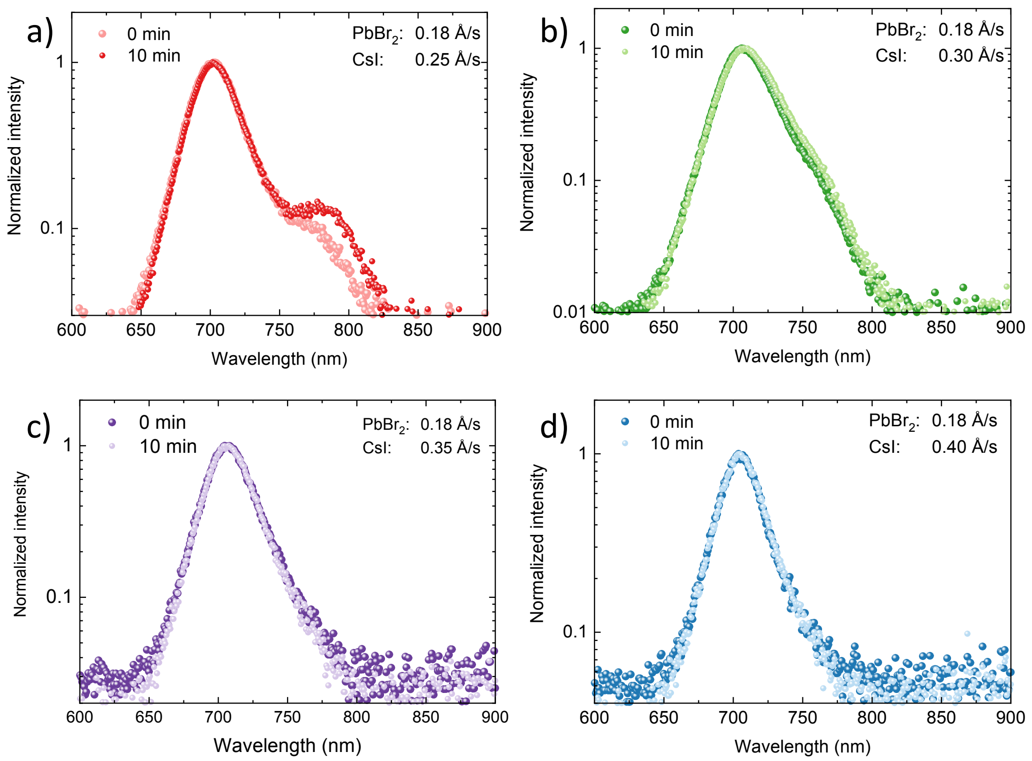

Figure S1. Photoluminescence (PL) spectra in semi-logarithmic scale under laser excitation (515 nm), at time 0 and after 10 minutes of continuous irradiation, for perovskite films with bandgap of approximately 1.75 eV (Br- content, $x = 0.27$) and increasing amounts of Cs+ (n), showing that the perovskite is photo-stable for CsI deposition rates > 0.3 Å/s.

In order to confirm that perovskite films obtained with CsI deposition rates > 0.3 Å/s are photostable, we have fit the PL spectrum in Figure S1c. The generalized Planck's law describes the radiation of a non-black body under a potential difference (quasi Fermi-level splitting, $\Delta\mu$) and reads in the Wien-approximation

$$I_{PL}(E) = \frac{2\pi}{h^3c^2}\frac{E^2\alpha(E)}{exp\left(\frac{E-\Delta\mu}{kT}\right)}$$

where E is the photon's energy, h is Planck's constant, c is the speed of light, $\alpha(E)$ is the absorptance of the semiconductor and kT is the thermal energy. From this, one can see that the slope of the PL is determined by the term $exp\left(-\frac{E}{kT}\right)$ in the low-wavelength region while it is governed by the decrease of absorptance below the bandgap in the high-wavelength region. Therefore, a PL spectrum is typically not symmetric. To show that there are no extra contributions of a low-energy phase we employed equation (1) to fit the PL spectrum of Fig. S1c using the

sensitive EQE spectrum of a perovskite solar cell with equivalent composition as the absorptance. To not be limited by the measurement noise starting at 750 nm, we extended the Urbach slope of the EQE down to 900 nm. The resulting theoretical spectrum assuming a temperature of 300 K is essentially identical to the measured PL. The small red-shift of 5 nm is likely due to a small discrepancy in the wavelength-calibration of the two measurement setups or to batch-to-batch variations in the perovskite deposition. Importantly, the quasi Fermi-level splitting does not influence the shape of the spectrum but just its intensity. We can hence conclude that we cannot see any additional phase from the PL spectra for perovskites obtained with CsI deposition rate > 0.3 Å/s.

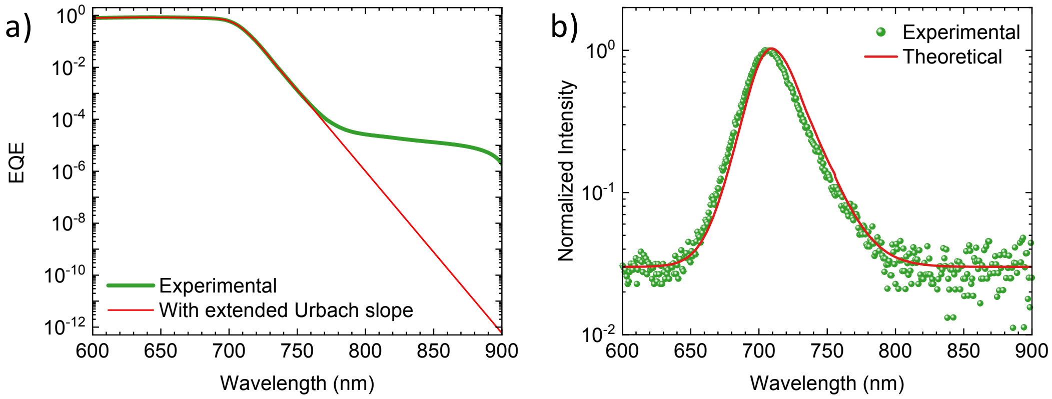

Figure S2. (a) EQE spectrum for a perovskite with bandgap of approximately 1.75 eV (Br^- content, x = 0.27) with and without the extended Urbach slope below the noise level. (b) Experimental and theoretical photoluminescence spectra for the same material, where the theoretical one is calculated using the EQE response with extended Urbach slope in (a) as the absorptance.

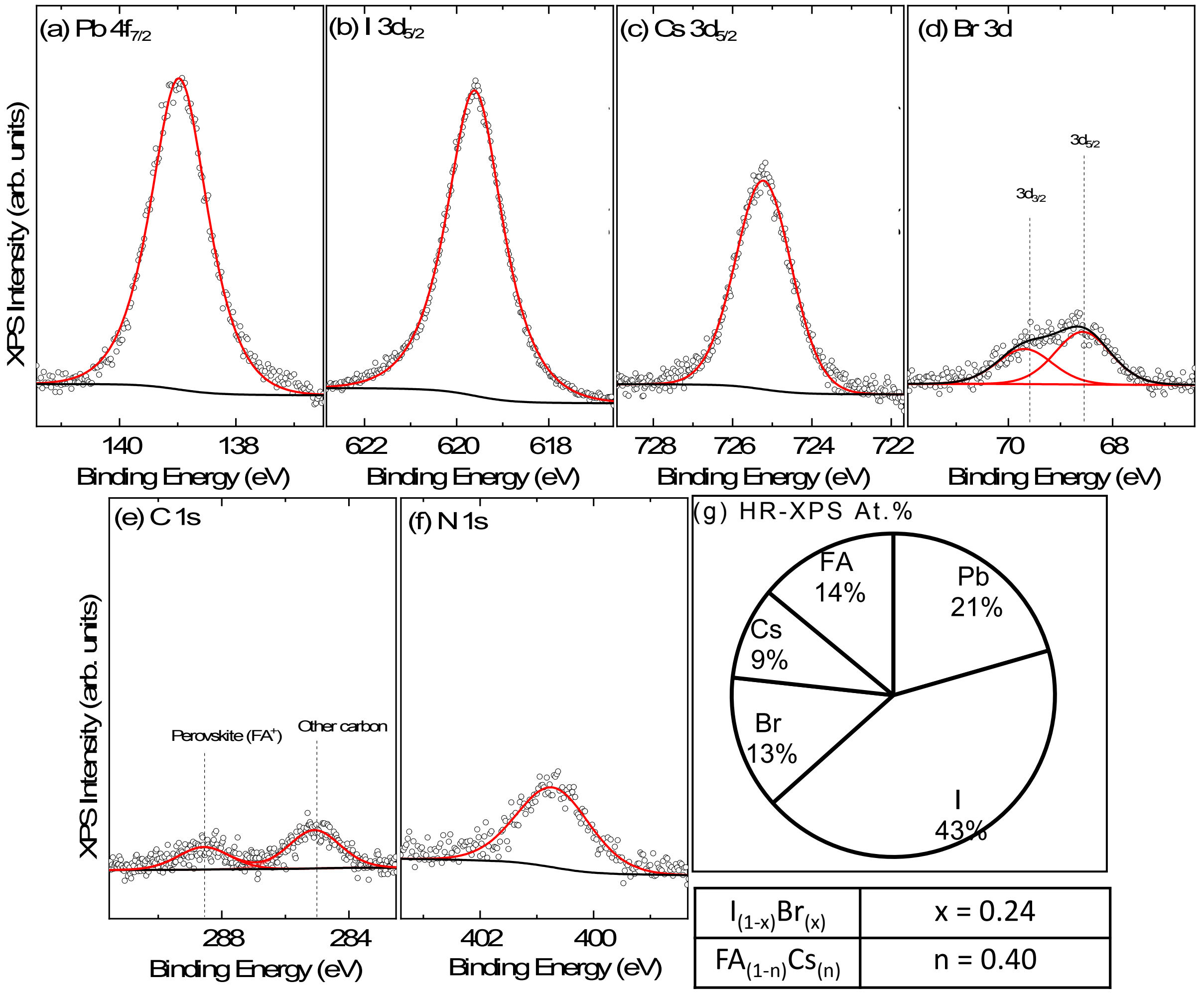

Figure S3. Top surface chemical composition. High resolution XPS spectra (Al−Kα = 1486.6 eV) of the perovskite film corresponding to core levels of (a) Pb-4$f_{7/2}$, (b) I-3$d_{5/2}$, (c) Cs-3$d_{5/2}$, (d) Br-3d, (e) C1s, (f) N-1s, and (g) atomic percentages (At.%) of the elements after normalization with corresponding elemental and spin-orbital splitting atomic sensitivity factors.

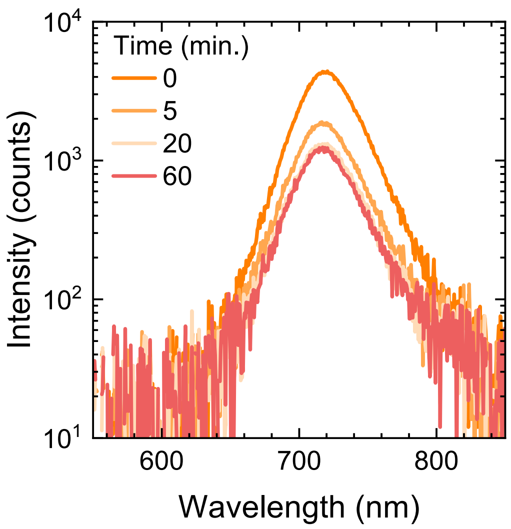

Figure S4. PL spectra recorded over time showing an initial decrease of the intensity which is found stable after 15-20 minutes of continuous irradiation.

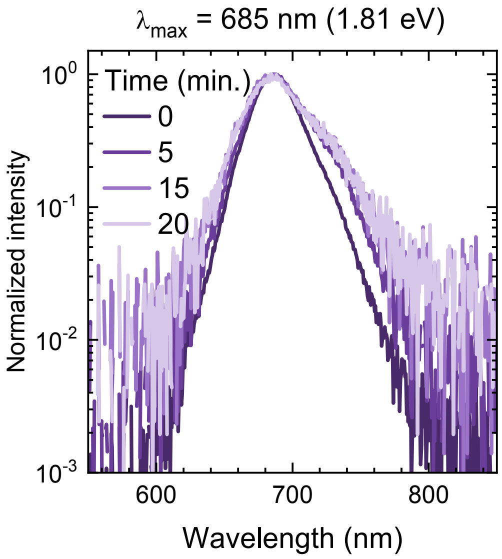

Figure S5. PL spectra recorded over time for a perovskite film of the type $FA_{0.5}Cs_{0.5}Pb(I_{1-x}Br_x)_3$ with bromide content $x = 0.4$.

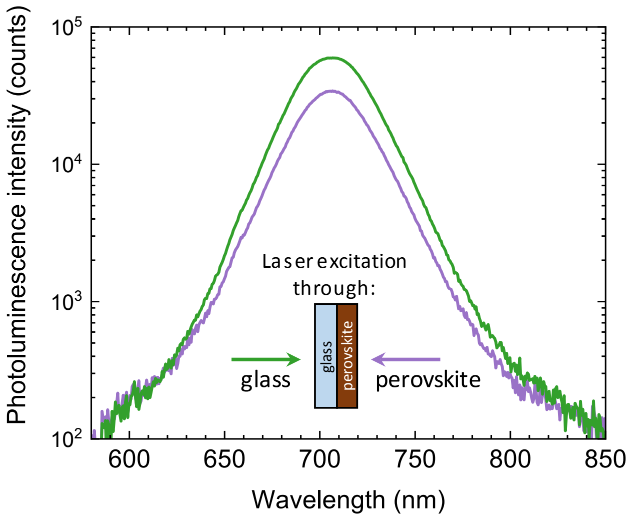

Figure S6. PL spectra for a perovskite with bandgap of 1.75 eV (Br^- content, x = 0.27) recorded by illuminating the sample through the glass substrate and through the perovskite surface.

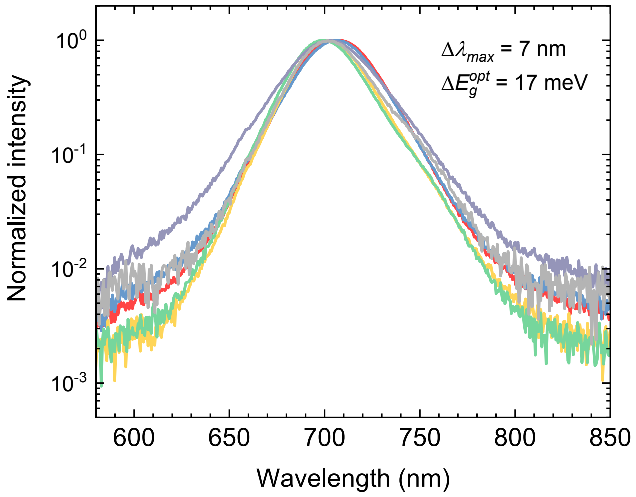

Figure S7. PL spectra for perovskite films with bandgap of 1.75 eV (Br^- content, x = 0.27) obtained from several consecutive deposition processes. The variation of the wavelength at maximum intensity (λ_{max}) is of 7 nm, corresponding to a bandgap fluctuation of only 17 meV.

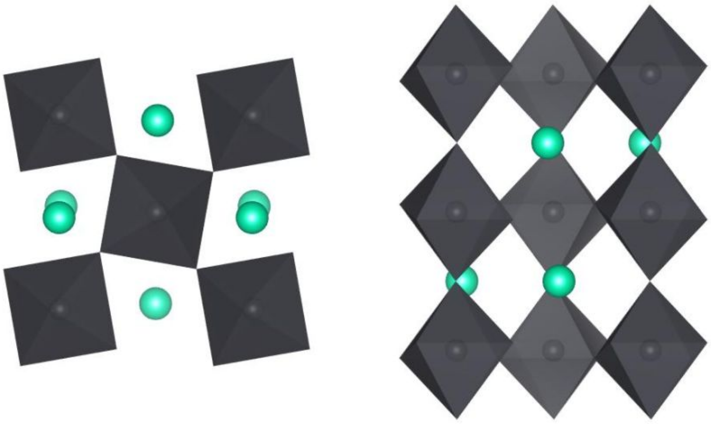

Figure S8. $CsPbBr_3$ crystal structure (Pnma space group) viewed along the b-axis (left) and c-axis (right). Green balls represent Cs^+ cations and grey octahedra represent $PbBr_6$ units.

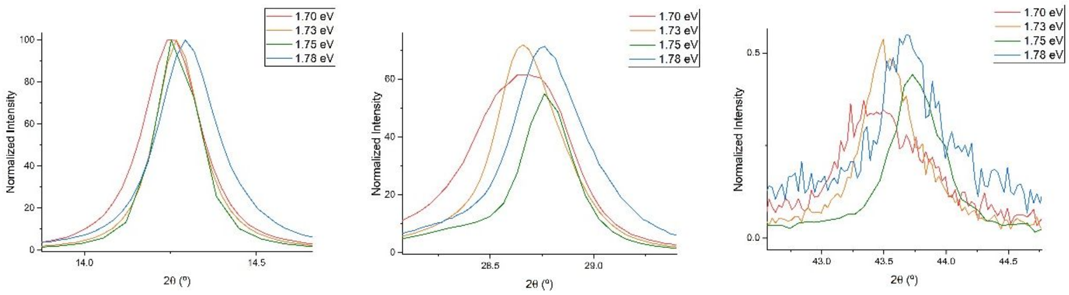

Figure S9. "Zoomed" view in some of the main XRD peaks. Samples corresponding to higher-bandgap compositions show diffraction peaks shifted to higher angles (i.e., smaller interatomic distances) as expected from the replacement of the bigger anion I^- with the smaller one Br^-. Note that unit cell volumes presented in Figure 2 are not simply derived from the direct observation of these signals but from whole-pattern deconvolution through Le Bail method, which yields a much more accurate result.

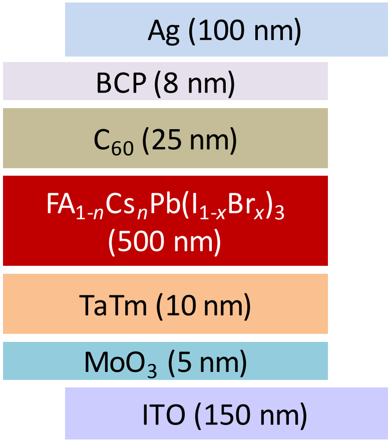

Figure S10. Schematics of the device layout used in this work.

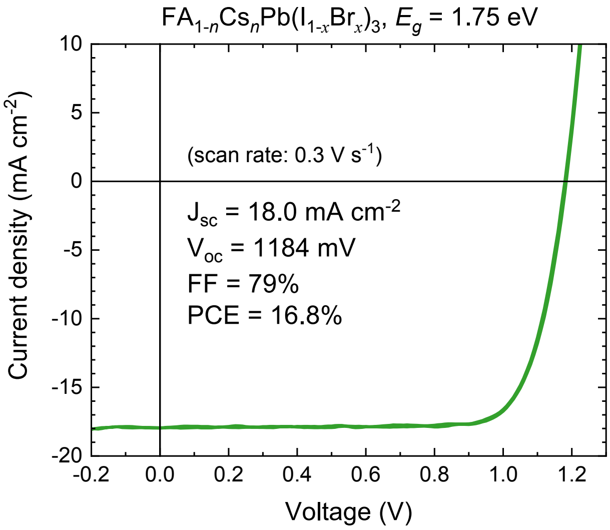

Figure S11. J-V curves under simulated solar illumination recorded in forward (from short to open circuit) and reverse (from open to short circuit) bias for the best solar cells using a 1.75 eV bandgap perovskite.

<u>Calculation of the bandgap, Urbach energy and the radiative limit of the V_{oc} from the sensitive EQE measurement</u>

The bandgap of an ideal semiconductor is defined as the energy where the absorption of light changes from 0 suddenly to 1. A real semiconductor has however not a sharp absorption onset but the absorption coefficient decays typically exponentially below the bandgap,

$$\alpha(E) = \alpha_0 \, exp\left(\frac{E}{E_U}\right), for\ E < E_g. \tag{1}$$

The slope of this decay is called the Urbach energy, E_U, and indicates the structural and thermic disorder in the absorber.[2,3] Here, we assumed that the EQE is proportional to the absorption coefficient and obtained E_U from a fit of the exponential decay of the EQE.

Furthermore, one can find an effective bandgap by looking at the derivative of the EQE. For the ideal semiconductor as in the Shockler-Queisser theory (SQ), this should be just a Dirac delta function at the bandgap, i.e. an infinitely high and infinitely narrow peak at the bandgap. Following Rau et al.,[4] $\frac{dEQE}{dE}$ can be interpreted as a distribution of SQ-bandgaps for a real semiconductor. It often looks similar to a Gaussian peak, so we extract the bandgap as the central energy of a Gaussian fit.

The radiative limit of the V_{oc} can be calculated with the measured J_{sc} and with the radiative limit of the dark saturation current, $J_{0,rad}$:[5]

$$V_{OC,rad} = kT\ . \tag{2}$$

This value includes the voltage losses because of difference between the measured J_{SC} and the theoretical possible value in the SQ theory as well as the radiative losses originating from the finite absorption tail.[4] To calculate the latter (corresponding to $J_{0,rad}$), we can use again the absorption tail of the EQE as a product with the black-body radiation at 300 K to calculate the luminescence spectrum at equilibrium (reciprocity relation) and integrate this:

$$J_{0,rad} = \int EQE(E)\varphi_{BB}(E{,}300\ K)dE. \tag{3}$$

In the SQ theory $J_{0,rad}$ is calculated by replacing the EQE spectrum with an step-function which yields lower values for $J_{0,rad}$ and thus a higher Voc.

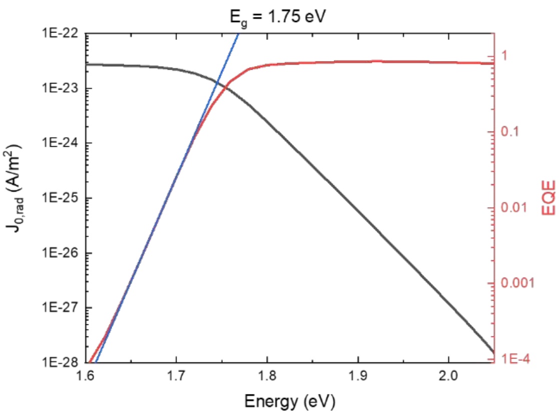

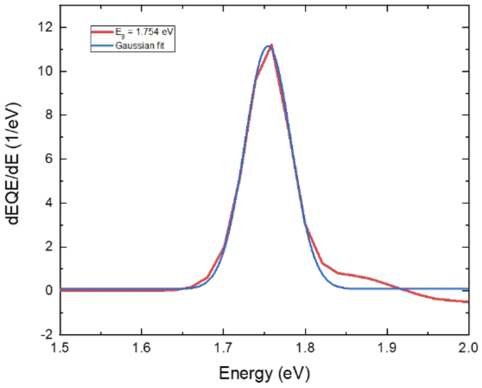

Figure S12. a) External quantum efficiency (EQE) spectrum (red) as in Fig. 4a for the perovskite with bandgap 1.75 eV with fit of the Urbach tail (blue) and integrated dark saturation current (black). b) Derivative of the EQE spectrum (red) with Gaussian fit (blue) to determine the bandgap.

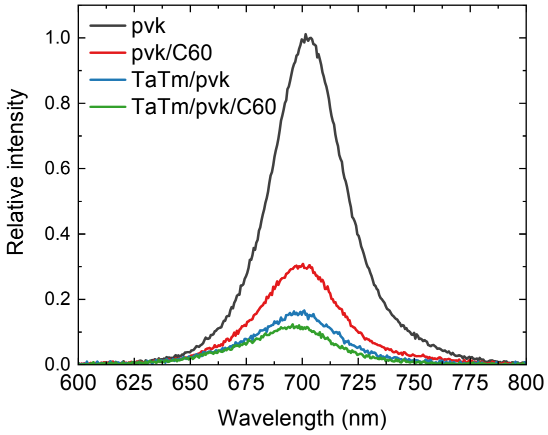

Sample	Relative PLQY	QFLS difference (eV)
pvk	1	0
pvk/C60	0.332	-0.028
TaTm/pvk	0.182	-0.044
TaTm/pvk/C60	0.138	-0.051

Figure S13. PL spectra of a perovskite film on glass, with and without the charge transport layers used in the solar cells. The relative PLQY for each variation is calculated by integration of the spectra and normalized to the one of the bare perovskite (PLQY =1). The corresponding QFLS difference calculated for each layout is also reported.

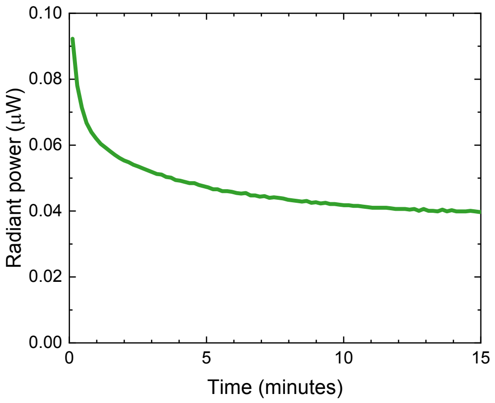

Figure S14. Time-dependent electroluminescence intensity for a perovskite solar cell in forward bias with a current density equivalent to its short circuit current.

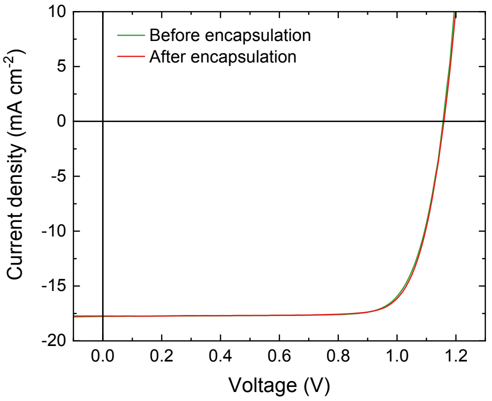

Figure S15. J-V curves for a wide-bandgap $FA_{0.65}Cs_{0.35}Pb(I_{0.73}Br_{0.27})_3$ perovskites solar cell with E_g = 1.75 eV before and after encapsulation.

References

(1) Scofield, J. H. Hartree-Slater Subshell Photoionization Cross-Sections at 1254 and 1487 EV. *J. Electron Spectros. Relat. Phenomena* **1976**, *8* (2), 129–137. https://doi.org/10.1016/0368-2048(76)80015-1.

(2) Urbach, F. The Long-Wavelength Edge of Photographic Sensitivity and of the Electronic Absorption of Solids. *Phys. Rev.* **1953**, *92* (5), 1324–1324. https://doi.org/10.1103/PhysRev.92.1324.

(3) Cody, G. D.; Tiedje, T.; Abeles, B.; Brooks, B.; Goldstein, Y. Disorder and the Optical-Absorption Edge of Hydrogenated Amorphous Silicon. *Phys. Rev. Lett.* **1981**, *47* (20), 1480–1483. https://doi.org/10.1103/PhysRevLett.47.1480.

(4) Rau, U.; Blank, B.; Müller, T. C. M.; Kirchartz, T. Efficiency Potential of Photovoltaic Materials and Devices Unveiled by Detailed-Balance Analysis. *Phys. Rev. Appl.* **2017**, *7* (4), 044016. https://doi.org/10.1103/PhysRevApplied.7.044016.

(5) Rau, U. Reciprocity Relation between Photovoltaic Quantum Efficiency and Electroluminescent Emission of Solar Cells. *Phys. Rev. B* **2007**, *76* (8), 085303. https://doi.org/10.1103/PhysRevB.76.085303.
